# Synchronized tapping facilitates learning sound sequences as indexed by the P300

**DOI:** 10.3389/fnhum.2014.00826

**Published:** 2014-10-29

**Authors:** Keiko S. Kamiyama, Kazuo Okanoya

**Affiliations:** ^1^Department of Life Sciences, Graduate School of Arts and Sciences, The University of TokyoTokyo, Japan; ^2^Emotional Information Joint Research Laboratory, RIKEN Brain Sceince InstituteSaitama, Japan; ^3^Okanoya Emotional Information Project, ERATO, Japan Science and Technology AgencySaitama, Japan

**Keywords:** synchronized tapping, event-related potentials, P300, mismatch negativity, musicians, auditory-motor network

## Abstract

The purpose of the present study was to determine whether and how single finger tapping in synchrony with sound sequences contributed to the auditory processing of them. The participants learned two unfamiliar sound sequences via different methods. In the tapping condition, they learned an auditory sequence while they tapped in synchrony with each sound onset. In the no tapping condition, they learned another sequence while they kept pressing a key until the sequence ended. After these learning sessions, we presented the two melodies again and recorded event-related potentials (ERPs). During the ERP recordings, 10% of the tones within each melody deviated from the original tones. An analysis of the grand average ERPs showed that deviant stimuli elicited a significant P300 in the tapping but not in the no-tapping condition. In addition, the significance of the P300 effect in the tapping condition increased as the participants showed highly synchronized tapping behavior during the learning sessions. These results indicated that single finger tapping promoted the conscious detection and evaluation of deviants within the learned sequences. The effect was related to individuals’ musical ability to coordinate their finger movements along with external auditory events.

## Introduction

The modulation of body movement in synchrony with external sensory inputs is a unique ability of humans and other animals that are capable of vocal learning (Hasegawa et al., 2010). In case of humans, even 5–24-month-old infants showed an immature ability to modulate their body movements to rhythmical sounds (Zentner and Eerola, 2010). Although human adults often show voluntary finger and foot tapping, and nod their head in synchrony with music, the reason why these movements are synchronized with auditory inputs is unclear. Indeed, Zentner and Eerola (2010) suggested that the propensity for coordinating auditory rhythmic pulses with movements facilitated the alignment of movement patterns with environmental and social sounds of adaptive significance. However, we assume that involuntary synchronization of body movements has another practical role, namely to facilitate the learning of auditory information. Also, along with Large and Jones (1999), synchronized tapping would contribute to increasing attention to auditory inputs. In this study, we examined the impact of single finger tapping on the memorization of sound sequences.

For that purpose, we first reviewed the past studies that showed the contributions of an auditory-motor network to “memory for performance.” Memory for performance—musicians’ memory that allows them to perform a specific musical piece (Palmer, 2006)—is an attractive example of multi-dimensional memory. It has at least two aspects: auditory and motor aspects. In previous studies, pianists exhibited involuntary activity in motor-related areas (involving the primary motor cortex) when they listened to well-known piano pieces (Haueisen and Knösche, 2001). Also when non-musicians listened to music which they were trained to play with the piano, neural activity was changed in motor-related areas (Lahav et al., 2005). Thus, it has been shown that the auditory-motor network was modulated by musical trainings and that the network came to voluntarily activate even when people just listen to music. In addition, training for a piano performance affects the auditory processing of the trained piece of music. We previously demonstrated that pitch deviations in a well-trained melody elicited larger error detection activity compared with melodies that were learned by simply listening (Kamiyama et al., 2010). We presented two melodies alternatively and asked participants to play the piano when one melody was presented. When the other melody was presented, they simply listened to it; they did not play any notes. After the learning sessions, we presented the melodies again and measured the participants’ brain activity with an electroencephalogram (EEG). During the event-related potential (ERP) recordings, 10% of the tones in these melodies deviated from the original tones. The deviant tones in the melody that the participants learned to perform elicited a larger mismatch negativity (MMN) compared with those in the other melody, indicating that the sensory-motor training promoted the memorization of the sound sequences and the detection of errors within them. We observed this effect only in the high absolute pitch group, which included those with a pronounced ability to identify a note without an external reference. In addition, Lappe et al. (2008) trained two groups of non-musicians over the course of 2 weeks. The participants in the sensory-motor-auditory (SA) group learned to play a musical sequence on the piano. Participants in the auditory (A) group listened to the music played by the participants in the SA group. A significantly larger MMN response was detected in the SA group than in the A group. Thus, sensorimotor-training caused plastic reorganizational changes in the auditory cortex.

As described above, the physical training of a piano performance activated the auditory-motor network and enhanced the auditory memory trace. However, several factors are included in the musical performance itself; one factor is the modulation of finger movement along with the intended musical production. Moreover, finger key tapping behaviors during a performance are constructed of each finger’s up-down movements and the sequential combination of them. Although it seems to be established that an actual piano performance, including sequential finger movements, facilitates the encoding of the auditory sequence (Lappe et al., 2008; Kamiyama et al., 2010), it has not been completely revealed how the activation of the auditory-motor network helps remembering melodies. For example, Lappe et al. (2008) suggested that the sensorimotor training promoted the changes in the auditory representation of the melodies, which lead to the enhancement of the MMN. They also suggested that the sensorimotor training caused more attentional resources to be spent on the perception of the auditory sequence, which led to the increased neural activity in the auditory system. According to their interpretation, actual piano performance would not be the only way to promote auditory processing. To confirm their opinion, the effect of other behaviors, such as simple tapping, on the auditory processing needs to be explored. We predicted that, like actual music performance, synchronized tapping would facilitate the memorization of the sound sequence, since the tapping behavior in synchrony with the sound onsets would recruit the auditory-motor network (Chen et al., 2006). In addition, we predicted the facilitation effect of tapping would vary with the individuals’ musical experiences or abilities. While the effect of sensorimotor-auditory training on auditory processing has been demonstrated even in musically naïve participants (Lappe et al., 2008, 2011), the effect was significantly correlated with a musical ability in musicians (Kamiyama et al., 2010). Only in musicians with a great ability to identify the sound frequencies along with the piano keys (i.e., absolute pitch), sensory-motor training with actual piano performance enhanced the auditory representations of the melody compared with passive listening. Therefore, in the present study, the individuals’ absolute pitch ability should also be considered so that we can clearly compare the synchronized tapping effect and actual piano performance effect on the auditory memory processing. In addition, the absolute pitch ability should be tested because it has been shown that this ability decreased P300 amplitudes (Klein et al., 1984). It was also suggested that synchronized tapping skill, which is supported by auditory-motor interactions, would be developed through implicit music exposure (Snyder et al., 2006) and explicit musical trainings (Ericsson et al., 1993; Repp and Doggett, 2007; Baer et al., 2013). In the present study, we focused on two ERP components, the auditory MMN and the P300 in order to assess auditory information processing.

The MMN is typically seen as a frontocentral negative ERP component that reflects unconscious error detection in sound repetition or sound sequences. The strength of an auditory memory trace of a frequently presented stimulus was developed during an oddball task, and an infrequent deviant stimulus elicited a MMN. It was reported that the MMN amplitude was affected by the presentation frequency of standard stimuli (Baldeweg et al., 2004; Haenschel et al., 2005), short-term discrimination trainings (Kraus et al., 1995), and long-term experience (Chandrasekaran et al., 2007). In addition, when information from multiple dimensions was available, a large amplitude MMN was observed (Schröger, 1998; Koelsch et al., 1999). Moreover, the MMN could be elicited by deviations from sequential rule patterns (Tervaniemi et al., 2001) and memorized sound sequences. In a previous study, we used the MMN as an index of the response to a deviation from learned sound sequences (Kamiyama et al., 2010).

The P300 is a positive ERP component that typically peaks 300 ms or more after the onset of task-relevant deviant stimuli (Duncan et al., 2009). Hence, the evaluation and conscious error detection processes would be reflected in the P300. P3a and P3b are subcomponents of the P300. However, P3a is elicited by task-irrelevant stimuli, is distributed across the midline frontocentral areas, and has an earlier peak latency than P300 (250–300 ms; Squires et al., 1975). The relation between the P3a and the P300 is still a matter of debate (Polich, 2007). Another P300 subcomponent, the P3b, is elicited by task-related stimuli and is distributed across the centroparietal areas. The task-related nature of the P300 has also been used to observe other ERP components. For instance, Paller et al. (1992) investigated whether the N400 component was sensitive to a deviation at the end of phrases excerpted from familiar musical pieces. Since they assumed that the N400 latencies would overlap with a P300-like positive component, named P340, they manipulated the positivity by using a discrimination task. In the absence of the task, the N400 changes were successfully observed. When participants performed the task, they indicated whether each melody ended with a proper note or not. In that particular study deviant tones elicited a larger P300 in the time window of 200–400 ms than standard tones. A smaller difference was observed when participants did not perform the discrimination task. Also, it has been reported that the P300 amplitude was affected by the strength of memory (working memory, and short-term and long-term memory) established during encoding, rehearsal methods, situational context updating during retrieval or a discrimination task, and memory load (Polich, 2007). Polich (1987) tested the sensitivity of the P300 (a positive component observed 250–400 ms after stimulus onset) to task difficulty, the frequency of deviant stimuli, and the interstimulus interval. The task difficulty was operated as difference of intensity between standard and target stimuli. Participants were instructed to discriminate tones presented at 40 (standard) and 60 (target) dB SPL in the easy task, while they discriminated tones presented at 40 (standard) and 45 (target) dB SPL in the hard intensity task. Independent of the stimulus deviation frequency, task difficulty affected the P300 amplitude and latency. Specifically, in the difficult task, a smaller amplitude of the P300 and a longer latency P300 was observed compared to the P300 amplitude observed in an easy task.

Thus, MMN and P300 reflect different stages of auditory information processing. Looking at both as an index for auditory deviance processing would contribute to examine the nature of tapping effect on auditory processing.

## Materials and methods

### Participants

Twenty Japanese amateur musicians (11 females) participated in this study. The mean age was 22.70 years (*range*: 18–47 years; standard deviation (*SD*): 7.26 years). Participants had at least 3 years of experience learning to play the piano (Table 1). On average, they started their piano training at 4.85 years of age (*range*: 3–10 years; *SD*: 1.93 years) and had 9.75 years of experience (*range*: 3–18 years; *SD*: 4.68 years). Five of them had studied singing or had played other musical instruments, such as violin, guitar, and flute. Including their piano musical experience and their experience with other musical instruments, on average, the continuous period of musical training was 10.16 years (*range*: 3–19 years; *SD*: 4.64 years). All participants had normal hearing and no history of neurological disease. All of the participants were right-handed. All procedures were approved in advance by the Ethics Committee of the Tokyo University. The participants gave written informed consent before the experiment.

**Table 1:** **Individual musical experiences, the percent correct (%) in an AP test, and synchronization errors in the learning session (only for the T condition)**.

	Piano training		Musical training	AP test score (%)	Variation of synchronization errors
	Onset (years old)	Period (years)	Period (years)
	3	18	18	100	53.63
	3	15	19	98.15	98.41
	3	15	15	99.07	41.08
	3	15	15	71.30	80.25
	3	14	14	40.74	62.67
	3	10	10	93.52	32.78
	3	4	12	100.00	40.17
	4	10	10	39.81	35.63
	4	3	3	36.11	46.19
	5	15	15	98.15	38.86
	5	14	14	91.67	32.71
	5	10	10	74.07	28.32
	5	6	6	96.30	55.78
	5	5	5	97.22	51.47
	6	9	9	46.30	49.75
	6	6	6	56.48	41.92
	6	4	4	53.70	41.19
	7	6	6	37.04	34.84
	8	11	11	66.67	45.80
	10	5	10	35.19	34.20
Average	4.85	9.75	10.6	71.57	47.28
SD	1.93	4.68	4.64	26.05	17.09

*Since all participants had received piano training prior to training with other musical instruments, the beginning age of general musical training was omitted from this table*.

### Procedure

#### Learning sessions

**Figure 1:**
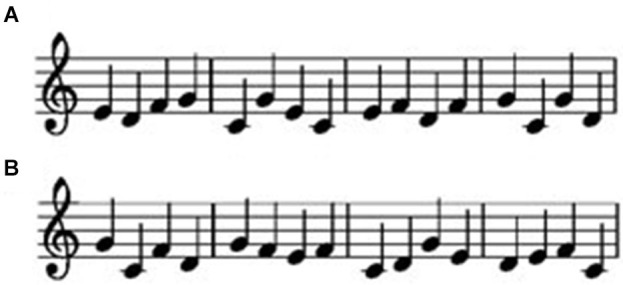
**Melodies A (A) and B (B)**.

For the learning sessions, we prepared two unfamiliar sound sequences (melody A and melody B; Figure 1). Each melody consisted of 16 notes; the following five piano tones were used: C4 (261.1 Hz), D4 (293.7 Hz), E4 (329.6 Hz), F4 (349.6 Hz), and G4 (392.0 Hz). Each note lasted approximately 750 ms, and each sequence lasted 12 s. Melodies A and B were presented via ear tubes, using a musical instrument digital interface (MIDI) program (Edirol SD90, Edirol). For the single finger-tapping task, we created a measurement instrument with two keys based on a response pad (NeuroScan, Inc.). We created this instrument so that participants could very lightly press the keys. The timing of each key tapping response and each tone presentation was recorded.

Participants sat on a reclining chair in a shielded room, wore an eye mask (Sleep eye mask; Concise, Inc.) and set a response keys on their lap. After they put on the eye mask, the learning session started. We presented melodies A and B alternatively, with an inter-trial interval (ITI) of 3000 ms. We asked participants to press the key at the beginning of one melody and held it down until the melody ended (NT condition). Also, when they listened to another melody, we asked them to tap the key with their right index finger in synchrony with the onset of each tone within the sound sequence (T condition). In one session 20 melodies were presented and, in total, two sessions were conducted. The assignment of melodies to each condition and the order of presentation were counterbalanced among participants. The tapping performances were recorded as text files using Scan 4.3 (SynAmps; NeuroScan, Inc.).

#### EEG recordings

Participants sat on a reclining chair in a shielded room, but did not wear the eye mask. We presented melodies A and B again during the EEG recordings. The difference between the training and EEG recording sessions were as follows: (1) within each melody, 10% of the tones were randomly chosen as deviant stimuli and were shifted up or down to the neighboring tones within the C major scale, using an online MIDI program. For example, D4 was infrequently changed to C4 or E4. (2) The duration of each tone varied between 740 ms and 940 ms to prevent alpha wave synchronization with the auditory stimuli. (3) The melodies were presented alternatively. Each melody was presented 20 times in every session. In total, three sessions were conducted. (4) We asked participants to attentively listen to the sound sequences without performing tapping behavior and to determine if there were any deviant tones within each melody. After each melody ended, they pressed one of two keys to indicate that they listened to the melodies. For half of the participants, the right key represented “all tones were correct” and the left represented “there was at least one error tone.” The other half of the participants pressed the left key when they found no error tones and the right key when they detected error tones. The main purpose of the task was to confirm that they carefully listened to melodies.

EEGs were recorded by a 64-channel Ag–Cl electrode cap (10–20 system) using the Scan 4.3 acquisition system (SynAmps; NeuroScan), with a 0.15–30 Hz band-pass filter and a sampling rate of 500 Hz. While a reference electrode was placed over the left mastoid during the data collection, electrodes on the bilateral earlobes were re-referenced on later off-line analysis. The impedances on all electrodes was measured and confirmed to be less than 5 kΩ. Vertical and horizontal electrooculograms (EOGs) were recorded simultaneously to eliminate the contamination of the EEG data by eye movements.

#### Absolute pitch (AP) test

After the EEG recordings, we tested the participants’ absolute pitch ability. To do so, we presented three octave piano tones (C3–H5, 36 piano tones) in a random order. Each tone was presented through ear tubes for 200 ms. Following each tone, participants judged which notes they heard by clicking the appropriate piano keys displayed on a personal computer monitor. For instance, when a C4 tone was presented, they were required to click a C4 key displayed on the computer. The next tone was presented 1000 ms after they answered. Each of the 36 tones was presented once each session. Three total sessions were conducted.

#### Questionnaire

At the end of the experimental procedures, we asked the participants about their musical experience. The main questions were when they started their musical training and how many years they continued the musical training.

### Analyses

#### AP test

In order to assess individuals’ ability to identify tone pitches, we calculated the percentage of correct responses (%) in the AP test across three sessions. Responses that differed from the correct tone by an octave or a semitone as presented tones were not counted as error responses. For example, when the D3 tone was presented, the correct responses were D4, D5, C#3, C#4, C#5, D#3, D#4, and D#5. We examined the relationship between the AP test scores and individuals’ musical experiences, using a correlational analysis. Additionally, we examined the correlation of the AP scores with the MMN and P300 amplitudes, in order to assess whether the ability to identify pitches affected the significance of error detection processes during the EEG recordings.

#### Tapping

In order to evaluate whether tapping during the learning sessions was synchronized with the tone onsets, the gaps between the timing of the tapping and the corresponding tone onsets (synchronization error, SE) were calculated in each trial and averaged across all trials in the T condition. Synchronization error reflects the temporal relationship between the stimulus and the action. When the SE value is positive, it indicates that the tapping onset lagged behind (Miyake et al., 2004). For each participant, we also calculated the SD of the SE as an index of variation of synchrony of tapping with auditory stimuli. The SE variation was used to determine if the degree of synchronization was correlated with the significance of the error detection activities, which were reflected in the MMN and P300 amplitudes. In addition, we used a correlational analysis to examine the relationship between the variation of tapping timing and individuals’ musical experiences.

#### EEG data

EEG data were analyzed using EDIT 4.3 (NeuroScan, Inc.). Event-related potential waveforms were time locked to the onset of each tone involving a 700-ms time period, including a 100-ms pre-stimulus baseline. To eliminate artifacts caused by eye movements, EEG data were rejected off-line whenever the amplitude exceeded ±100 µV. If the behavioral responses were incorrect, the EEG data for these trials were excluded from further analysis. In addition, two trials immediately after deviant notes, and a trial in which C4 or G4 was expected were also excluded. The remaining data (42.47%, 42.71%, 35.63%, and 38.25% of the standard tones in the T condition, deviant tones in the T condition, standard tones in the NT condition, and deviant tones in the NT condition, respectively) were averaged for each stimulus type (standard or deviant tone) and condition (T or NT condition). In order to account for the topographical distribution of the ERPs in the statistical analysis, the scalp surface was divided into seven topographical regions (Abla et al., 2008; Abla and Okanoya, 2009; Kamiyama et al., 2010); each region corresponded to three electrodes: middle anterior (Fp1, Fz, and Fp2), middle central (FCz, Cz, and CPz), middle posterior (P3, Pz, and P4), left anterior (F3, FC3, and F7), right anterior (F4, FC4, and F8), left posterior (CP3, TP7, and P7), and right posterior (CP4, TP8, and P8).

To evaluate the MMN and P300 components, the mean amplitude at 170–210 ms and 300–450 ms after stimulus onset were analyzed by analysis of variance (ANOVA) (αlevel = 0.05). The time window for measuring the MMN was defined as between 20 ms before and 20 ms after the peak amplitude in the grand average waveform (Otten et al., 2000). As for the P300, because the latency of the peak amplitude was not identified from the individual ERP waveforms, the time window was determined by visual inspection. The mean amplitude data were subjected to a repeated measures ANOVA with condition (T and NT conditions), stimulus type (standard and deviant stimuli), and electrode site (seven regions of interest (ROIs)) as within-subject factors. A Bonferroni correction of the *p*-values was used in all of the *post hoc* tests.

For subsequent analyses, the mean amplitude within the time window of 170–210 ms for the MMN and 300–450 ms for the P300 were averaged across the FCz, Cz, and CPz electrodes for each stimulus type. Using the MMN and P300 amplitude in middle central area, we calculated the impact of a single finger tapping on error detection or evaluation activities as follows: [(deviant tones in T condition−standard tones in T condition)]−[(deviant tones in NT condition−standard tones in NT condition)]. In order to focus on how synchronized tapping would influence auditory memory encoding and the later auditory error processing, the relationships between SE of tapping and MMN amplitude, as well as between SE of tapping and P300 amplitude were assessed by correlation analyses. We also examined the correlation between these amplitudes and the AP test scores. The purpose of this analysis was to compare how actual piano performance (Kamiyama et al., 2010) and synchronized tapping would affect the auditory memory processing.

### Hypothesis

We anticipated the following results: (1) the deviant stimuli within each sound sequence would elicit a MMN and a P300 regardless of the learning conditions; (2) synchronized tapping would facilitate sound sequence learning, and the MMN and P300 amplitude would be larger in the T than in the NT condition; and (3) musical experience and ability would enhance the effect of tapping on sound sequence learning.

## Results

### AP test

The average AP test score was 71.57% (*range*: 35.19–100%; *SD*: 26.05%; Table 1). In order to assess the relationship between AP test scores and individual’s musical experiences, in particular, when they started musical training and how long they continued the piano training or training with the other musical instruments, we conducted correlation analyses. The earlier the participants started musical training, the higher were their AP test scores (*r* = 0.48, *p* < 0.05). The longer they experienced musical training the higher were their AP scores (*r* = 0.47, *p* < 0.05). When the participants’ experience with the other instruments were excluded, there was no relationship between the continuous period of piano training and their AP scores (*r* = 0.41, *p* = 0.07).

### Tapping

To assess tapping accuracy, we calculated SE, the gap between each instance of tapping and the onset of the corresponding tone (Table 1). The mean SE was −71.57 ms (*SD* = 36.76) indicating that, on average, the tapping movements proceeded the tones. On average, the variation of the SE was 47.28 (*SD* = 17.09) and was not correlated with any of the musical experiences (starting age of musical training: *r* = −0.39, *p* = 0.08; continuous period of piano training: *r* = 0.37, *p* = 0.11; continuous period of musical training: *r* = 0.43, *p* = 0.06).

### Behavioral performance

The percent correct for both the T and the NT conditions, in the behavioral test during the EEG recordings exceeded 90% (Table 2). Accuracy was not significantly affected by conditions (paired *t*-test: *t*_(19)_ = 0.75, *p* = 0.46). In addition, for both conditions, there was no significant difference between the rate at which the participants incorrectly found error notes within the correct sequences and the rate at which they did not find any errors within the deviant sequences (paired *t*-test: T condition: *t*_(19)_ = 1.34, *p* = 0.20; NT condition: *t*_(19)_ = 0.94, *p* = 0.36).

**Table 2:** **The behavioral test responses during the EEG recordings**.

	T condition				NT condition			
	Hits	False		No response	Hits	False		No response
		“error”	“no error”			“error”	“no error”
mean (%)	93.25	1.67	4.42	0.33	94.00	2.25	3.58	0.25
SD	8.56	2.29	8.36	0.87	7.50	2.77	6.50	0.83

*The data are expressed as percentages. “Hits” indicates that participants correctly found error tones. “False” indicates that they incorrectly found error tones (“error”) or no error tones (“no error”). “No response” means that they failed to press any keys after each melody was presented*.

### ERPs

#### MMN

**Figure 2:**
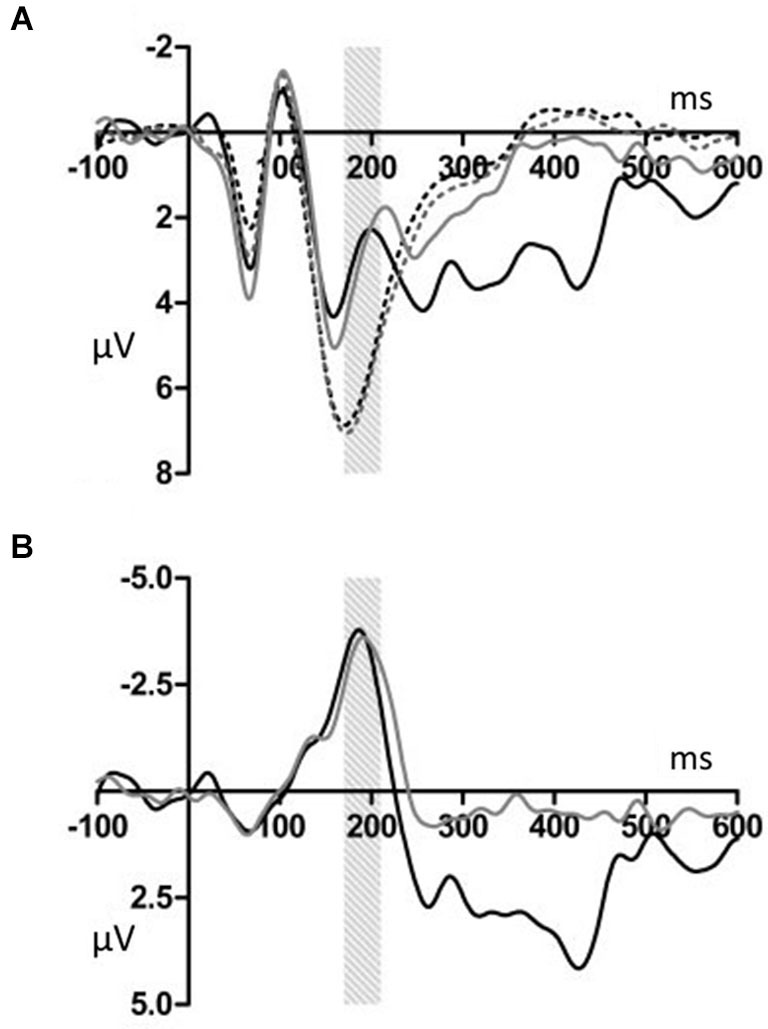
**(A) Grand average ERPs for electrodes FCz**. The deviant notes elicited larger amplitude negative component around 190 ms in the T and NT conditions. The black dotted line indicates the response to the standard stimuli in the T condition. The black solid line indicates the response to the deviant stimuli in the T condition. The gray dotted line indicates the response to the standard stimuli in the NT condition. The gray solid line indicates the response to the deviant stimuli in the NT condition. **(B)** The differential wave of standard and deviant tones observed at FCz. The black indicates the response in the T condition. The gray line indicates the response in the NT condition.

**Figure 3:**
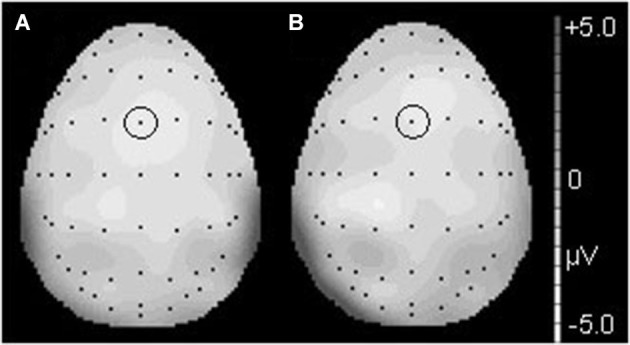
**The topographical differences response to the deviant and correct tones at 186 ms in the T condition (A) and at 190 ms in the NT condition (B)**. The black dots represent each electrode site. The black dot within the black circle represents electrode FCz.

Figures 2, 3 show that, for both the T and NT conditions, the MMN component was observed around the FCz electrode; its amplitude was not affected by conditions. A repeated-measures ANOVA with condition, stimulus type, and ROI as within-subjects factors was conducted, using the mean ERP amplitude at 170–210 ms after each tone onset. The ANOVA showed a significant main effect of stimulus type (*F*_(1,19)_ = 29.99, *p* < 0.001, *power* > 0.99) and an interaction between ROI and stimulus type (*F*_(6,144)_ = 7.27, *p* < 0.001, *power* > 0.99). *Post hoc* test showed that deviant tones elicited significantly larger negativity than standard tones at all electrode sites (middle anterior: *F*_(1,19)_ = 34.76, *p* < 0.001, *power* > 0.99; middle central: *F*_(1,19)_ = 25.22, *p* < 0.001, *power* > 0.99; middle posterior: *F*_(1,19)_ = 19.27, *p* < 0.001, *power* = 0.99; left anterior: *F*_(1,19)_ = 30.94, *p* < 0.001, *power* = 0.99; right anterior: *F*_(1,19)_ = 26.40, *p* < 0.001, *power* > 0.99; left posterior: *F*_(1,19)_ = 20.44, *p* < 0.001, *power* = 0.99; right posterior: *F*_(1,19)_ = 18.28, *p* < 0.001, *power* = 0.98). These results indicated that the MMN was induced by deviant tones within learned melodies and was widely distributed across the scalp irrespective of the tapping conditions used in the learning sessions. This interpretation was supported by the finding of no interaction effects including the within-subject factor, condition (condition × stimulus type: *F*_(1,19)_ = 0.11, *p* = 0.74, *power* = 0.06; condition × stimulus type × ROI: *F*_(6,114)_ = 0.21, *p* = 0.97, *power* = 0.10).

**Figure 4:**
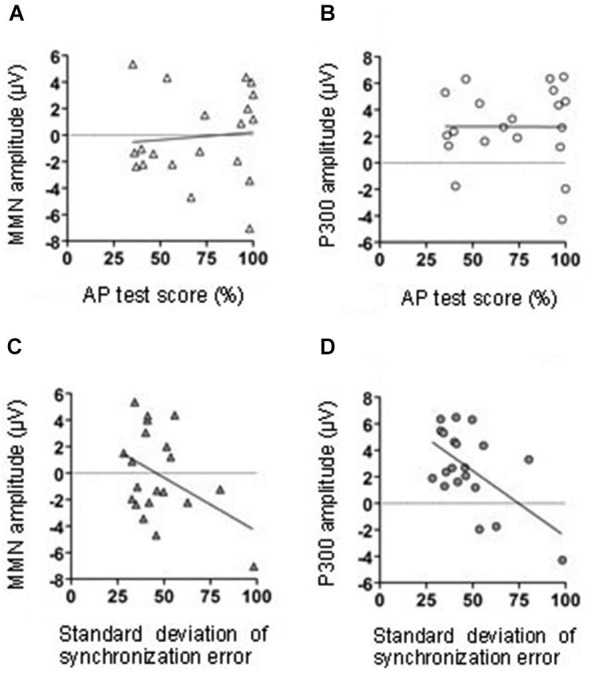
**The relationship between MMN amplitude and AP test score (A)**. The relationship between the P300 amplitude and AP test score **(B)**. The relationship between MMN amplitude and tapping synchrony **(C)**. The relationship between the P300 amplitude and tapping synchrony **(D)**.

Correlational analyses were conducted to assess the relationship between the MMN amplitude in the middle central area (FCz, Cz, and CPz), AP test score, and tapping performance. Neither AP test score nor the variation of the SE were significantly correlated with the MMN amplitude (AP test score: *r* = 0.09, *p* = 0.71; tapping variation: *r* = − 0.42, *p* = 0.07; Figures 4A,C, respectively).

#### P300

**Figure 5:**
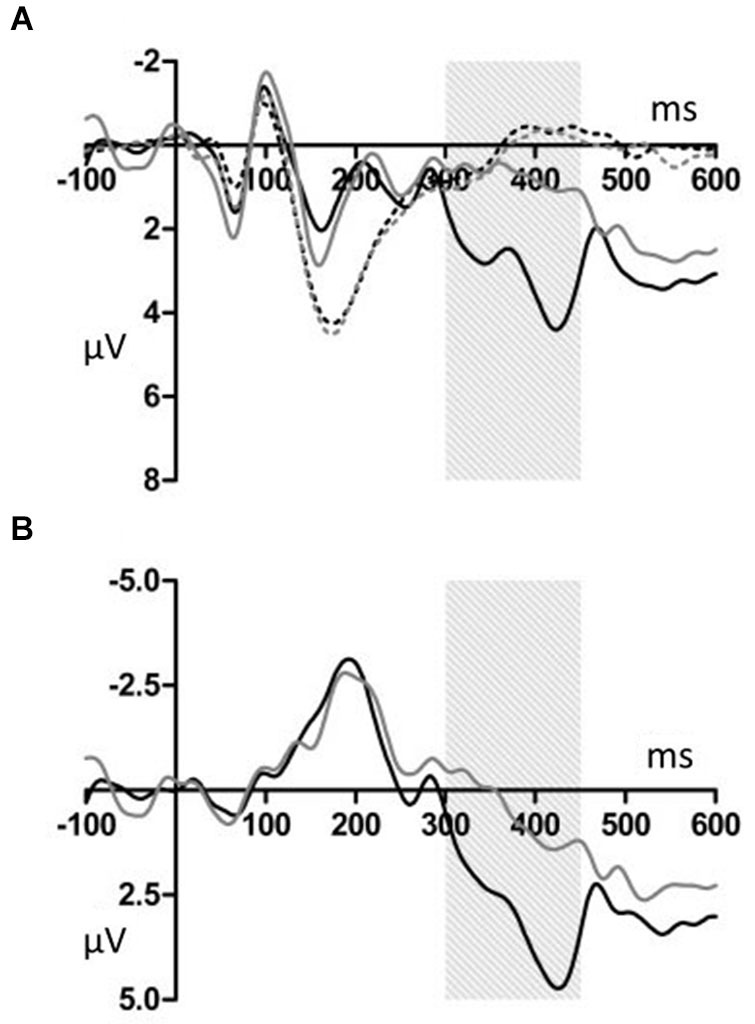
**(A) Grand average ERPs for electrodes CPz**. The deviant notes elicited larger amplitude negative components at 300–450 ms only in the T condition. The black dotted line indicates the response to the standard stimuli in the T condition. The black solid line indicates the response to the deviant stimuli in the T condition. The gray dotted line indicates the response to the standard stimuli in the NT condition. The gray solid line indicates the response to the deviant stimuli in the NT condition. **(B)** The differential wave of standard and deviant tones at CPz. The black line indicates the response in the T condition. The gray line indicates the response in the NT condition.

**Figure 6:**
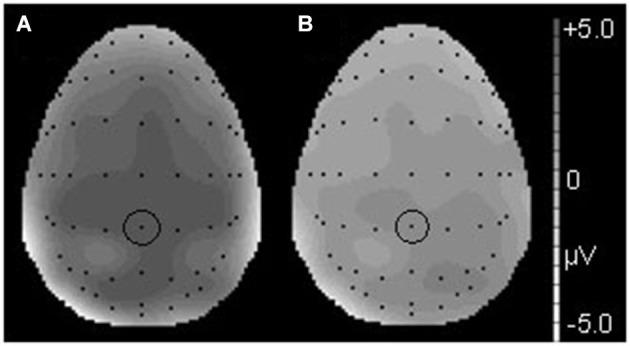
**The topographical differences in response to the deviant and correct tones at 426 ms in the T condition (A) and at 436 ms in the NT condition (B)**. The black dots represent each electrode site. The black dot within the black circle represents electrode CPz.

Deviant tones elicited a P300 in the T but not in the NT condition (Figures 5, 6). A repeated measures ANOVA with condition, stimulus type, and ROI as within-subject factors showed that the mean amplitude within 300–450 ms varied with stimulus type (*F*_(1,19)_ = 13.32, *p* < 0.01, *power* = 0.80), supporting the observation that the deviant stimuli elicited the P300 component. Since the interaction between stimulus type and ROI was significant (*F*_(6,144)_ = 6.98, *p* < 0.001, *power* > 0.99), we conducted additional ANOVAs at each ROI. It was revealed that deviant tones elicited larger positive potentials in centroparietal areas (middle anterior: *F*_(1,19)_ = 0.24, *p* = 0.63, *power* = 0.08; middle central: *F*_(1,19)_ = 24.18, *p* < 0.001, *power* > 0.99; middle posterior: *F*_(1,19)_ = 25.57, *p* < 0.001, *power* > 0.99; left anterior: *F*_(1,19)_ = 5.09, *p* < 0.05, *power* = 0.57; right anterior: *F*_(1,19)_ = 1.47, *p* = 0.24, *power* = 0.21; left posterior: *F*_(1,19)_ = 17.60, *p* < 0.001, *power* = 0.98; right posterior: *F*_(1,19)_ = 20.10, *p* < 0.001, *power* = 0.99). In addition, since the interaction between condition and stimulus type was significant (*F*_(1,19)_ = 14.00, *p* < 0.005, *power* > 0.94), the stimulus type effect for each condition was examined. The analysis revealed that the deviant tones elicited large amplitude P300s in the T condition (*F*_(1,19)_ = 26.49, *p* < 0.001,* power* = 0.99) but not in the NT condition (*F*_(1,19)_ = 0.02, *p* = 0.89, *power* = 0.05). The condition × stimulus type × ROI interaction was not significant (*F*_(6,114)_ = 1.59, *p* = 0.16, *power* = 0.59).

We conducted a correlation analysis to determine the relationship between the P300 effect in the middle central area (FCz, Cz, and CPz), AP test scores, and tapping performance. The variations in synchronized tapping performance (SE) were negatively correlated with the P300 amplitude (*r* = −0.58, *p* < 0.01). Thus, with the decreasing variation of synchronized tapping in learning sessions, the difference of P300 amplitudes in T and NT conditions significantly increased (Figure 6D). The AP test scores were not significantly correlated with the P300 amplitude (*r* = −0.01, *p* = 0.98; Figure 6B).

## Discussion

In order to assess how single finger tapping in synchrony with sound sequences influences the memorization of the auditory inputs, we measured the electrophysiological activity of musicians. Participants listened to two melodies alternatively with synchronized finger tapping and without synchronized finger tapping. After these learning sessions, we presented the same two melodies again and measured the participants’ EEG. Because the tones infrequently deviated from the original tones, these deviant stimuli elicited an MMN and a P300, reflecting error detection and evaluation processing. Although the MMN was observed in both the tapping and the no-tapping conditions (Figures 2, 3), significant P300 effect was observed only in the tapping condition (Figures 5, 6). These results indicated that the earlier error detection was not affected by synchronized tapping while listening to the learned melodies. On the other hand, the tapping promoted the later evaluation process for the detected errors. Additionally, the P300 amplitude was negatively correlated with the synchronization errors in the tapping task (Figure 4D), indicating that the effect of synchronized tapping was supported by an audio-motor network that was developed by the longitudinal musical trainings.

### Tapping task was correctly performed

Before examining the effect of tapping on the error detection processes, we assessed whether the tapping was correctly performed. The instances of tapping preceded the tone onsets by 71.57 ms. The antecedence of tapping is called “negative asynchrony” (Dunlap, 1910) and is often observed in synchronized tapping. The standard deviation of the SE varied greatly between participants and ranged from 28.32 to 98.41 (Table 1). However, because none of them exceeded 98.55 (the average plus three SDs), we concluded that there were no outliers. Consequently, we concluded that our participants could correctly perform the synchronized tapping.

### Single finger tapping did not modulate the automatic error detection process

As we predicted, in both the T and the NT conditions, the MMN was elicited by the deviant tones. However, the MMN amplitude did not significantly differ between these conditions (Figures 2, 3). Thus, the finding indicates that synchronized tapping did not improve sound sequence learning and automatic error detection during the subsequent EEG recordings. In Kamiyama et al. (2010), sensory-motor training with piano performance enhanced the auditory representation of performed melody and error detection, which were indexed by the MMN amplitude. Moreover, several studies reported that actual piano performance training supported the auditory-motor interaction when the participants listening to the trained sound sequences (Lahav et al., 2005; Chen et al., 2012). The reason why these previous findings were not replicated in this study could be that our task was relatively easy and passive. The combination of tactile and auditory feedback might be important for the learning efficiency. Playing and listening, on the other hand, differ in that the former involves active and volitional motor acts that generate tones whereas in the latter case tones are merely registered. In our tapping task, participants have an accompanying role and the volitional motor act might be reduced to a rather mechanical process. Additionally, learning to play the piano is not merely a more difficult task involving the translation of visual cues from the notation and requiring fine auditory discrimination. Sensorimotor interaction is also important, because when playing the piano, movements are naturally paired with auditory feedback. The previous studies (Bangert and Altenmüller, 2003; Lappe et al., 2008, 2013; Kamiyama et al., 2010) support the assumption that all these factors play a crucial role to achieve plasticity effects in the human brain. An additional explanation is that our tapping task was somewhat more a synchronization task and thus produced no coupling between a hand movement and a certain pitch. The result indicated that synchronization task we used did not have significant effect on changing the auditory representations.

### Single finger tapping enhanced the evaluation process

We observed that the P300 was elicited by deviant stimuli in the T but not in the NT condition (Figures 5, 6). The latency and distribution of the P300 indicated that we observed typical P3b component (Duncan et al., 2009). Therefore it was ensured that the P300 component was induced by task-relevant deviant stimuli. The P300 reflects attention allocation, context updating, and fluctuations in arousal state (Polich and Kok, 1995). The P300 amplitude is small in difficult tasks (Polich, 1987). Therefore, our results indicated that synchronized tapping, which occurs concurrently with learning sound sequences, facilitated attracting the participants’ attention and promoted the evaluation process when the participants listened to them. This interpretation is consistent with a theory of attentional dynamics which was suggested by Large and Jones (1999). According to them, when being faced with everyday events, people are engaged by temporally patterned changes occasioned by natural forces. Such events comprise actions and movements related to their temporal characteristics. Large and Jones (1999) called the behavior of internal oscillations the “attending rhythms.” The attending rhythms allow us to entrain to external events and target attentional energy at expected points in time. In the present study, since these melodies were given in a fixed tempo, participants might be easily engaged by their temporal pattern and caused a great attention and expectation toward tones. Additionally, it is possible that the synchronized tapping behavior promoted further attention toward these tones. Thus, the increased attention toward the melody might lead to enhanced encoding of auditory processing in learning sessions and to an enlarged P300 effect in EEG recording sessions.

However, this idea is contrary to the behavioral test performance. The participants showed equally accurate performance in T and NT conditions (Table 2). If conscious processing was enhanced in the T but not the NT condition, the difference should have been reflected in the behavioral test results. There are two possible explanations. Firstly, the task itself might have been too easy for our participants so that their performance might show a ceiling effect. Secondly, it is plausible that there were some deviations within each melody that were easy to find. Since we required the participants to determine if a sound sequence included at least one deviant note, the participants could easily answer “yes” if only one salient deviation was detected within a series of sounds. In addition, we assumed that a deviation from a C or a G note (i.e., H or A notes), might be easy to detect because neither H nor A notes were presented in the training sessions. Thus, they may have functioned as novel sound elements in the EEG recording sessions. Therefore, the P300 in the T condition might reflect the evaluation of deviant tones within the sound elements included in the learned sequences, whereas the behavior test reflected all error detection processes including that for finding novel tones. Since the main purpose of the behavioral task was to confirm that the participants attended to the auditory stimuli, the inconsistency between the ERP and the behavioral performance results would not be a major problem here. Hence it is reasonable to conclude here that synchronized tapping indeed facilitated the memorization of the sound sequence, according to a previous finding that P300 amplitude changes were related to initial encoding (see Polich, 2012).

### Absolute pitch ability showed little impact on the tapping effect on learning

Since pitch test score was positively related to the onset and continuous period of their musical trainings, the AP test scores might indirectly reflect individuals’ musical experiences. We predicted that the ability to identify sound frequencies would be related to the significance of the tapping effect on sound sequence learning. However, the AP test score was not significantly correlated with either the MMN or the P300 amplitude (Figures 4A,B). In order to explain this result, we suggest two possibilities. Firstly, the deviations within the sound sequences used in the present study might have been too easy to be recognized and the AP score effect was too small to be detected. However, in our previous study, we found that the MMN effect was related to the performance in the AP test (Kamiyama et al., 2010). In our previous study we used the same auditory stimuli as those used in the present study. The memory trace of the sound sequences was enhanced by performing the sequences with the piano only in musicians with high absolute pitch ability; it was not enhanced in musicians without absolute pitch. Therefore it was self-evident to test the impact of individuals’ musical experience or musical ability also in this study with similar auditory stimuli. The single finger tapping, however, affected musical sequence learning in a way that was only remotely related to the absolute pitch ability and musical experiences. It has been suggested that people have an innate ability to synchronize with external sounds. In support of this idea, even 5–24-month-old infants showed an immature ability to modulate their body movements to rhythmical sounds (Zentner and Eerola, 2010). In adults, the behavior can be often observed as spontaneous foot tapping and finger tapping to music. Although the tapping ability could be largely affected by their musical training (Aoki et al., 2005; Baer et al., 2013), it could be acquired to some degree without special musical training. This may not be true for the acquisition of absolute pitch ability. Thus, it is implied that musical experience and absolute pitch ability were independent of the tapping effect on sound sequence learning.

### Accuracy of tapping was related to the tapping effect on learning

We also examined the relationship between the significance of the tapping effect on sound sequence learning and individual’s musical ability represented by synchronized tapping performance. As the participants accurately tapped in synchrony with the sound onsets, the tapping behavior caused great attentional resources toward tones and, thus, enhanced the P300 effect (Figure 4D). Therefore, it is possible that the ability to modulate finger movements in synchrony with auditory inputs could serve as a facilitator for learning them. Also we have to consider the confounding variable, for instance, the ability to focus attention on the tones influences both tapping accuracy and auditory processing. According to Tierney and Kraus (2013), the synchronized tapping relates to sustained attention. This possibility should be tested in future studies.

Here, we need to consider that there was no significant correlation between tapping performance and musical experiences. Previous studies showed that musicians could maintain synchronized tapping without paced stimuli more accurately (Baer et al., 2013) and could modulate tapping timings more flexibly than non-musicians (Repp and Doggett, 2007). These findings clearly showed that long-term musical training improved the ability to synchronize with external auditory inputs. In addition, even for musicians with a high level of expertise, the amount of deliberate practice correlated strongly with several tests of pure technical ability including finger tapping (Ericsson et al., 1993; see also Sloboda, 2000). Their findings indicated that tapping performance would be improved by musical trainings, although this effect was not observed in the present study. One possible explanation is that our participants were all musicians who practiced musical instruments for at least 3 years and we did not control the duration of piano training and that of other musical instruments.

### Future perspectives

In general, a target for future study is to continue to identify the aspects of musical experience or ability that are responsible for the facilitation effect on sound sequence learning. Although no significant correlation was observed between tapping performance and musical experience factors (e.g., the age of onset of musical trainings and the continuous period of them), the results of previous studies indicated that musical training should improve tapping skills (Ericsson et al., 1993; Repp and Doggett, 2007; Baer et al., 2013). Therefore, whether the stability of tapping affected the facilitation effect independently of individual’s musical training history needs to be tested in future studies. For that purpose, musically naïve participants should be examined, too. In order to make a clear contrast with our previous study (Kamiyama et al., 2010), here we examined the synchronized tapping effect only in skilled amateur musicians with an experimental procedure based on that study. However, leaning to tap to a short melody might be a more challenging task, as well as a more efficient way to cause the sensorimotor-auditory effects on auditory processing, in non-musicians than in musicians. Hence, in line with the sensorimotor-auditory training effect studies, further complementary approach would be required to understand differences in learning strategies between non musicians and musicians.

## Conclusion

Our study is novel in that we examined the effect of synchronized tapping on auditory memory processing. We found that the synchronized tapping promoted the later evaluation process for the detected errors, as indexed by the enhanced amplitude of the P300. The P300 effect was negatively correlated with synchronization errors in the tapping task, indicating that the effect of synchronized tapping was supported by the audio-motor network, which was developed by the longitudinal implicit or explicit musical trainings. These findings shed light on the benefit of expertise to modulate body movement along with external auditory inputs.

## Author contribution

Role of each author in the study: Keiko S. Kamiyama Formulated initial planning; performed data collection and the statistical analyses; contributed to data assessment and interpretation; prepared the manuscript draft and Kazuo Okanoya Formulated initial planning; contributed to data assessment and interpretation; performed critical review of multiple drafts of the manuscript.

## Conflict of interest statement

The authors declare that the research was conducted in the absence of any commercial or financial relationships that could be construed as a potential conflict of interest.
